# Effectiveness and Safety of Treating Negative Emotions after PCI from the Perspective of Qi and Blood: A Systematic Review and Meta-Analysis

**DOI:** 10.1155/2022/8604472

**Published:** 2022-09-08

**Authors:** Fang Liu, Fuming Liu, Jia Li, Yuying Du, Xu Han

**Affiliations:** ^1^The First Clinical Medical College, Nanjing University of Chinese Medicine, Nanjing, China; ^2^Department of Endocrinology, Affiliated Hospital of Nanjing University of Chinese Medicine, Xuzhou Hospital of Chinese Medicine, Xuzhou, China; ^3^Department of Cardiology, Affiliated Hospital of Nanjing University of Chinese Medicine, Jiangsu Province Hospital of Chinese Medicine, Nanjing, China; ^4^Department of Rehabilitation, Changzhou Dean Hospital, Changzhou, China; ^5^Department of Geriatrics, Affiliated Hospital of Nanjing University of Chinese Medicine, Jiangsu Province Hospital of Chinese Medicine, Nanjing, China

## Abstract

**Background:**

Many patients undergoing PCI have been reported to suffer from psychological distress and negative emotions. Several lines of evidence have indicated that PCI patients with negative emotions are particularly vulnerable to myocardial reperfusion injury when they face psychological challenges. As proven by clinical trials and research, traditional Chinese medicine (TCM) has certain advantages in alleviating psychological symptoms in PCI patients. The level of evidence for TCM is not yet high. There is no existing systematic review to evaluate the effectiveness and safety of TCM in post-PCI patients.

**Methods:**

PubMed, EMBASE, Cochrane Library, Web of Science, CNKI, VIP, Wanfang Database, and CBM were searched to identify randomized controlled trials (RCTs) that treated negative emotions after PCI using qi and blood cotherapy. The search period was from database inception to March 1, 2022. After applying the inclusion and exclusion criteria to the RCTs, research quality evaluation and data extraction were conducted, and a meta-analysis of the articles was performed using Revman 5.3 and Stata 12.1.

**Results:**

A total of 14 RCTs involving 1314 patients were included. Meta-analysis results indicated that compared with the anti-anxiety and depression medications group, the qi-blood harmony group was more effective in clinical outcomes (RR = 1.19, 95% CI (1.13, 1.25), *P* < 0.00001), maintaining the stability of angina pectoris (SMD = 0.65, 95% CI (0.29, 1.01), *P*=0.0004), increasing the degree of patients' satisfaction (SMD = 0.95, 95% CI (0.25, 1.65), *P*=0.008), reducing the frequency of attacks (SMD = 0.64, 95% CI (0.11, 1.18), *P*=0.02), and the incidence of adverse reactions (RR = 0.54, 95% CI (0.43, 0.68), *P* < 0.00001). The HAMA and HAMD scores were significantly lower after treatment, and there was no significant difference between the two groups (HAMA: RR = 1.03, 95% CI (0.95, 1.12), *P*=0.4). The efficacy of the two groups was comparable. In terms of reduction of the HAMD score, after sensitivity analysis, the literature by Liang was found to be significantly influencing the results, and after excluding the results of Liang, the qi-blood harmony group was superior to the Western medicine group for reducing the HAMD score (RR = 1.12, 95% CI (1.01, 1.24), *P* < 0.05), which was significantly different. The results of this review, combined with the grade evaluation, suggest that the HAMA posttreatment score reduction, the efficacy of TCM treatment, and the incidence of adverse reactions were supported by moderate evidence, and the HAMD posttreatment score reduction and the SAQ score were supported by low-quality evidence.

**Conclusion:**

Treating negative emotions after PCI based on the idea of “harmonization of qi and blood” can effectively improve the clinical efficacy, the stability of angina pectoris and the degree of patient satisfaction, and can reduce the frequency of angina attacks and the incidence of adverse events. There was no significant difference between the two groups for reductions in the HAMA and HAMD scores. However, more high-quality, large sample, multicentre RCTs are still needed for further verification. *PROSPERO Registration Number*: PROSPERO CRD42022313169.

## 1. Introduction

Coronary heart disease (CHD) is one of the deadliest diseases worldwide [1] and it threatens global public health. As the main treatment measure, percutaneous coronary intervention (PCI) has the advantages of less trauma, shorter hospital stays, and significant efficacy and is currently one of the important means of treating CHD in China and has been widely used in clinical practice. Although this technique is relatively mature, it only addresses the “symptoms” and does not fundamentally address or reverse the underlying etiology of coronary atherosclerosis, and increasing evidence suggests that CHD is associated with bicardial diseases [2], such as stress, anxiety, and depression [3]. Psychological issues have been shown to be an independent risk factor for cardiovascular disease, increasing the risk of major adverse cardiovascular event (MACE) recurrence, increasing the number of readmissions and recurrences, and affecting the outcome of PCI procedures to some extent. With the development of the “Bicentric Medicine” model, the risk of MACEs was found to be increased 6.21-fold in patients with CHD combined with moderate anxiety and 4.32-fold in those with moderate depression in a study conducted 1 year after coronary intervention [2]. Therefore, the patient's postoperative mental health has become an important part of the recovery from PCI.

Currently, Western drugs such as selective 5-hydroxytryptamine reuptake inhibitors (SSRIs), benzodiazepines (BDZ), and compound preparations (Deanxit) have been shown to be effective in reducing psychological symptoms in patients, but they are a “double-edged sword.” Patients may experience different degrees of side effects after taking these drugs, such as gastrointestinal reactions, neurological reactions, hepatic and renal decompensation, drug dependence, and rebound from drug withdrawal. Meanwhile, combining these drugs with cardiovascular drugs may cause drug-drug interactions and affect their efficacy. Some patients are intolerant and refuse to take their prescribed medication. Therefore, the tradeoff of how to treat PCI postoperative bicardial disease is a major challenge that clinicians must face.

Psychotherapy is also a means of improving patients' postoperative mood disorders, but it requires long-term patient cooperation and is expensive, difficult to see any efficacy in a short period, and is often prone to patient drop-out. Traditional Chinese medicine (TCM) has a long history, and it has a good foundation for the public because it has fewer side effects and it focuses on overall regulation. Qi to the sky of Su Wen says that “Internal and external harmony, then the evil cannot harm,” and most doctors believe that the disease is located in the “heart” and “liver.” The core of the disease is “Qi and blood disharmony,” based on the idea of the “holistic view” of TCM; as the saying goes, “The mind and body are in harmony, the Qi and blood are in harmony, the Yin is calm and the Yang is secret, then the spirit is cured.” Qi-blood cotherapy emphasizes the treatment of heart diseases by tonifying Qi, invigorating blood, and clearing phlegm while at the same time considering the release of emotions, the regulation of Qi, and the reassurance of the mind, both in a two-pronged way. The available evidence has supported the potential benefit of TCM for postoperative patients. However, the strength of evidence for individual RCTs is relatively weak, and most of them are using empirical and self-prepared formulas, coupled with the wide variety of Chinese medicines, inconsistent dosage forms, and doses. Some Chinese medicine ingredients have not undergone rigorous pharmacological mechanism of action studies, so the safety and efficacy of TCM are not yet supported by scientific evidence. In this article, we used meta-analysis to systematically evaluate the effectiveness and safety of treating negative emotions after PCI with TCM, aiming to provide a scientific basis for clinical decision-making.

## 2. Methods

### 2.1. Protocol and Registration

The protocol of this systematic review and meta-analysis was based on the Preferred Reporting Items for Systematic Reviews and Meta-Analyses Protocol (PRISMA-P) [4]. Moreover, the protocol for this study has been registered with the International Prospective Register of Systematic Reviews (PROSPERO, CRD42022313169).

### 2.2. Literature Search

**Figure 1:**
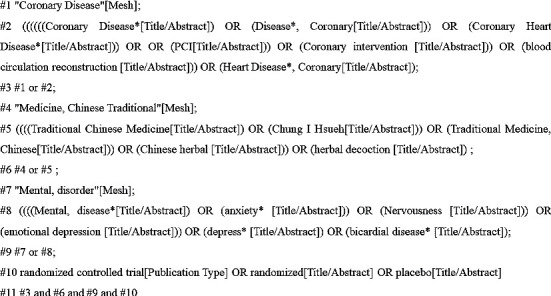
Search strategy.

Predefined search strategies were used to identify relevant clinical trials. We searched the following eight databases: PubMed, EMBASE, Cochrane Library, Web of Science, CNKI (China National Knowledge Infrastructure), VIP database, Wanfang database, and CBM (Chinese Biomedical Database). The retrieval time was from their inception to March 1, 2022. The search was conducted using a combination of subject terms and free words. The Chinese search terms included coronary disease, PCI, coronary intervention, Chinese medicine, qi-blood cotherapy, bicardial diseases, anxiety, depression, etc.; English search terms included coronary disease, PCI, coronary intervention, traditional Chinese medicine, negative emotions, bicardial diseases, randomized controlled trial, etc. The retrieved documents were imported into Endnote X9 for unified management. Taking PubMed as an example, the specific search strategy is shown in Figure 1.

### 2.3. Inclusion and Exclusion Criteria of the Literature

Studies were selected according to the Cochrane Handbook for Systematic Reviews of Interventions [5]. The inclusion criteria for the meta-analysis were as follows: (1) Study type: clinical randomized controlled trial (RCT), whether or not blinded or allocation concealment was performed. Limited to Chinese and English literature. (2) Subjects: patients who met the diagnostic criteria of CHD [6] and had successfully undergone PCI, regardless of age, sex, region, and race, and met the World Health Organization's diagnostic criteria for depression with an anxiety disorder (ICD-10). The diagnostic criteria for TCM evidence were referred to as the diagnostic points of depression in the “Guidelines for the Treatment of Common Diseases in Chinese Internal Medicine—TCM Evidence Section” [7] (TCD code: BNX020). (3) Intervention: based on conventional CHD drug treatment, the experimental group used the formula of qi-blood cotreatment (benefitting Qi to nourish the heart, invigorating blood to dispel phlegm, and combining the effects of draining the liver and relieving depression, unlimited water decoction, granules, pills, etc.), and the control group used anti-anxiety and depression drugs such as deanxit and escitalopram. (4) Outcome indicators: primary outcome indicators: efficacy of the TCM, HAMA, and HAMD posttreatment score reductions; secondary outcome indicators: Seattle Angina Questionnaire scores (SAQ) and incidence of adverse events.

### 2.4. Efficacy Determination Criteria

The reduction of HAMA and HAMD scores after treatment was calculated according to the efficacy assessment criteria developed by the Psychiatric Rating Scale Manual [8]. Reduction rate = (pretreatment score − posttreatment score)/(pretreatment score) × 100%.

If the symptoms and signs basically disappeared, with a scale reduction rate >75%, it was a clinical cure; if the symptoms and signs improved significantly, with a 75% ≥ scale reduction rate >50%, it was effective; if the symptoms and signs improved and had less influence on daily life, with a 50%≤ scale reduction rate >25, it was somewhat effective; if the symptoms and signs did not improve significantly or worsened, with a scale reduction rate ≤25%, it was ineffective. Total clinical efficiency rate = ((number of cured + number of effective + number of effective)/total number of cases) × 100%.

The efficacy index of TCM was calculated with reference to the criteria established in the Guidelines for Clinical Research on Guiding Principles for Clinical Research of New Chinese Medicines [9].

Chinese medical evidence efficacy index (*n*) = (pretreatment score − posttreatment score)/pretreatment score × 100%.

Significant improvement in symptoms and signs and a ≥70% decrease in points was considered effective; improvement in symptoms and signs and a 70% to ≥30% decrease in points was considered somewhat effective; and no significant improvement in symptoms and signs or even aggravation and a ≤30% decrease in points was considered ineffective.

Total clinical effectiveness rate = ((number of effective cases + number of effective cases)/total number of cases) × 100%.

Meanwhile, we excluded studies with the following features: animal experiments, systematic evaluations, reviews, empirical, and medical cases; other therapies, such as acupuncture, Ba Duan Jin, and acupressure, were part of the intervention; the outcome indicators did not include the observed outcome indicators in this study; duplicate published literature; and full text not available.

### 2.5. Literature Screening and Data Extraction

Literature screening was conducted independently by two researchers. First, the titles and abstracts of the studies were read to initially screen out those that did not meet the criteria, and for those that were uncertain in the initial screening, a secondary screening was conducted according to the inclusion and exclusion criteria after reading the full text. In case of disagreement, a third party participated in the discussion and assisted in the judgment.

The data extraction included basic information of the included literature, such as the name of the first author and the year of publication; basic characteristics of the study population, such as sample size and age; interventions and treatment courses; elements of the risk of bias evaluation; and outcome indicators.

### 2.6. Quality Assessment

Using the Cochrane Handbook 5.1.0 manual as the standard [10], the risk of bias evaluation included selection bias (random sequence generation and allocation concealment), implementation bias (blinding of the investigators and subjects), measurement bias (blinded evaluation of the study results), follow-up bias (completeness of outcome data), reporting bias (selective reporting of research results), and other biases. Each of the above entries was judged as “low risk, unclear, high risk,” and any disagreement could be decided by mutual discussion or consultation with a third party.

### 2.7. Data Synthesis and Statistical Analysis

Meta-analysis of the included literature data was performed using Review Manager 5.3 and Stata 12.1 software. Heterogeneity was analyzed using the *χ*^2^ test (test level *α* = 0.10), and if the heterogeneity between studies was more acceptable (*P* > 0.10 and *I*^2^ ≤ 50%), a fixed effects model was selected for calculation; otherwise, a random-effects model was chosen. The dichotomous variables were subjected to meta-analysis of their outcome indicators using relative risk (RR) as an effect size indicator. The level of meta-analysis was set at *α* = 0.05. When significant heterogeneity existed, subgroup analysis or sensitivity analysis was performed to find the source of heterogeneity, or only descriptive analysis was performed, depending on the data available. Funnel plots were used to analyze whether there was publication bias in the included studies.

### 2.8. Quality of Evidence Grade Evaluation

GRADEprofiler 3.6 was applied to evaluate the outcome indicators, and the evidence levels were provided as high, moderate, low, and very low.

## 3. Results

### 3.1. Selected Studies and Characteristics

**Figure 2:**
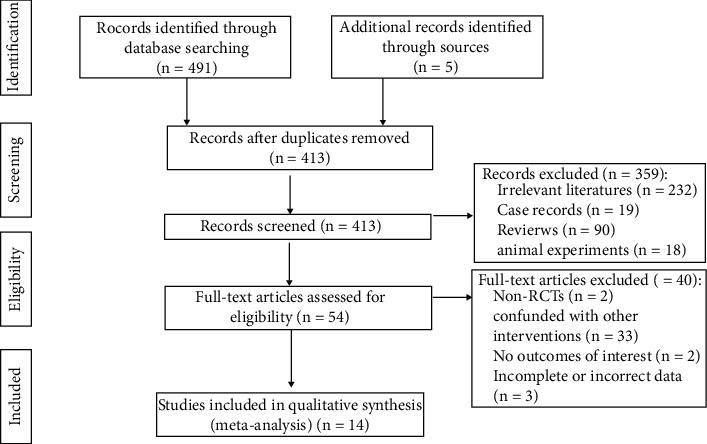
Flow diagram of the included studies. RCT, randomized controlled trial.

In this study, a total of 496 studies were retrieved, 83 duplicates were excluded, 359 studies were excluded after reading the titles and abstracts, 40 studies were further excluded after intensive full-text rescreening, and 14 studies were finally included, all of which were in Chinese. The screening flow chart is shown in Figure 2.

### 3.2. Basic Characteristics of the Literature

Fourteen studies were included in this study, involving 1314 patients, 655 in the test group and 659 in the control group, with a sample size of 30 to 151 cases in the individual studies. The interventions in the test group were heart-liver cotreatment formulas based on conventional drug therapy for CHD. Conventional drug therapy included dual inhibition of platelet aggregation, lipid-lowering, plaque stabilization, and other drugs to treat the underlying disease (antihypertensive, hypoglycemic, heart rate slowing), and myocardial nutrition. The control group was given anxiolytic-depressant medication on top of conventional CHD medication, which was increased or decreased as appropriate based on the patient's symptoms. The duration of treatment ranged from 2 to 16 weeks. The basic characteristics of the 14 studies are detailed in Table 1.

### 3.3. Literature Bias and Quality Assessment

**Figure 3:**
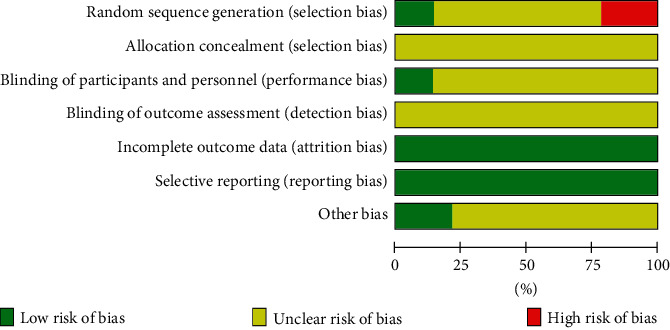
Risk-of-bias graph.

The quality of the included RCTs is shown in Figure 3. For the 14 included studies, all mentioned a randomized method of grouping, among which two studies [11, 14] used a randomized number table method and were considered “low risk”; two studies [20, 21] grouped patients according to their wishes; and one study [16] grouped patients according to the order in which they were seen and they were assessed as “high risk”; the remaining studies only involved “randomization” without specifying the protocol. For sequence allocation concealment, none of the studies mentioned it. Two studies [14, 17] described the use of blinding, and none of the others were described as blinded. Eleven [11–13, 15–17, 20–24] studies reported adverse effects. No study explicitly described whether the outcome evaluators were blinded. All studies included a balanced population at baseline, with good data completeness and perceived low risk of selective reporting bias. Three studies [14, 17, 23] were supported by government funding programs, had no conflicts of interest and were otherwise assessed as “low risk” of bias; the source of the other bias for the remaining trials was unclear. The risk of bias for each study is shown in Figure 3.

### 3.4. Meta-Analysis Results

#### 3.4.1. HAMA Pre- and Posttreatment Score Reduction

**Figure 4:**
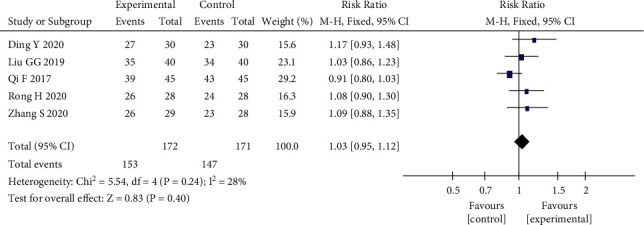
Forest plot of the HAMA scale posttreatment reduction.

Five RCTs [12, 14,1 7, 20, 21] used the posttreatment reduction in the HAMA scale as an outcome indicator. The results showed that *P*=0.24 > 0.1, *I*^2^ = 28% < 50%, mild heterogeneity among studies, and the fixed effects model was selected for analysis, which finally yielded RR = 1.03, 95% CI (0.95, 1.12), *P*=0.40, and *Z* = 0.83 (see Figure 4), which showed that there was no significant difference in the reduction of HAMA scale scores in the qi and blood cotherapy herbal medicine group compared with the Western medicine group. These results showed that there was no significant difference in the reduction of HAMA scale scores between the two groups, and the efficacy of both treatments was comparable. When sensitivity analysis was performed, each included study was excluded one by one, and it was found that *I*^2^ decreased from 28% to 0% after excluding the study by Qi [14], *P*=0.1. It was found that the other four studies all used Chinese herbal decoctions, and the study by Qi used granules, suggesting that the different dosage forms may have some effect on the heterogeneity.

#### 3.4.2. HAMD Posttreatment Score Reduction

**Figure 5:**
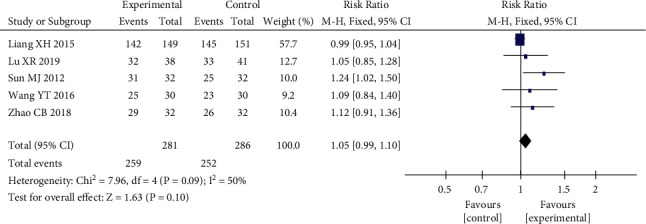
Forest plot of HAMD scale posttreatment reduction.

**Figure 6:**
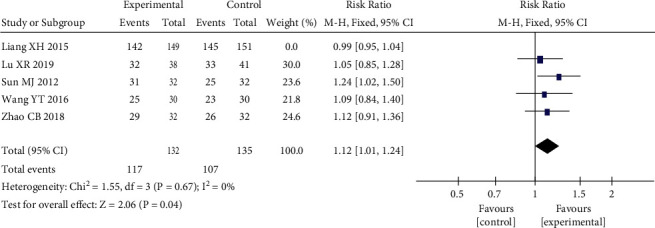
Forest plot of HAMD scale posttreatment reduction excluding Liang XH.

**Figure 7:**
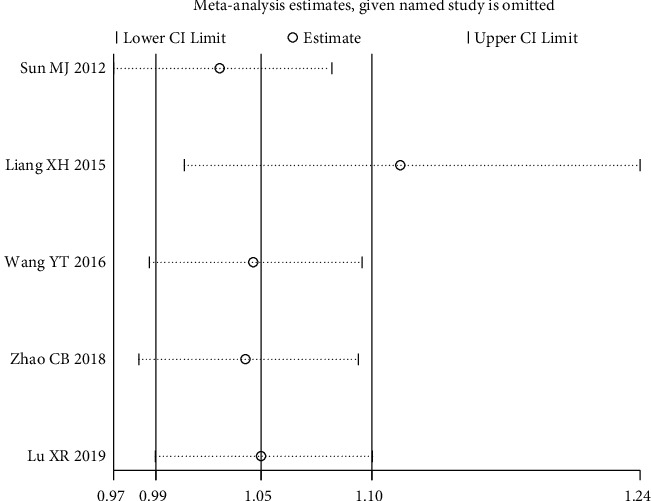
HAMD scale posttreatment reduction sensitivity analysis.

Five RCTs [13, 15, 19, 22, 24] used the HAMD scale posttreatment score reduction as an outcome indicator. The results showed that there was moderate heterogeneity among studies with *P*=0.09 < 0.1, *I*^2^ = 50%, RR = 1.05, 95% CI (0.99, 1.10), *P*=0.1. The results showed that there was no statistically significant difference in the reduction of HAMD scale scores in the heart-liver cotherapy herbal group compared with the Western medicine group, and the efficacy of both was comparable, as shown in Figure 5. Sensitivity analysis was performed on five studies, excluding each included study one by one, and found that the study of Liang [15] significantly affected the stability of the trial results. The *I*^2^ decreased from 50% to 0% after its exclusion, *P*=0.04 < 0.05, and the meta-analysis showed a large change in effect size (see Figure 6). Sensitivity analysis using STATA found that the point estimates for the results of Liang [15] fell outside the 95% CI for the combined effect size (see Figure 7). This study had a large effect on heterogeneity with its relatively large sample size, and after its exclusion, the heart-liver cotherapy herbal group was statistically superior to the Western medicine group in effectively reducing the HAMD scale scores (RR = 1.12, 95% CI (1.01, 1.24), *P* < 0.05).

### 3.5. Efficacy of TCM

**Figure 8:**
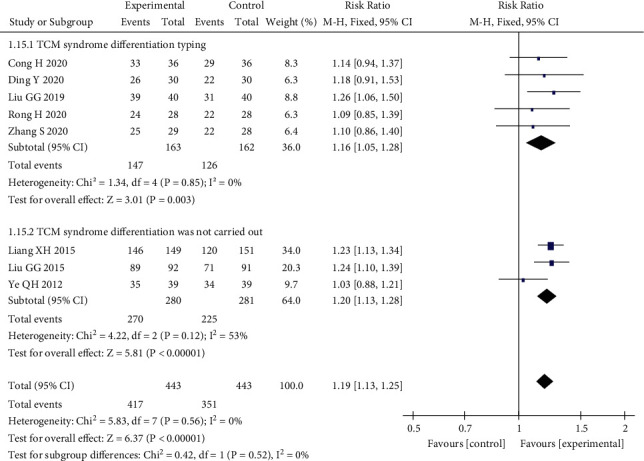
Forest plot of subgroup analysis of the efficacy of TCM.

Eight studies [11, 12, 15–18, 20, 21] compared the clinical efficacy of the qi-blood approach with that of antidepressant and anxiety medication. Symptoms included irritability, depression, chest pain, fatigue, palpitations, insomnia, dry mouth, forgetfulness, sighing, heat in the five hearts, etc. The included studies were subjected to subgroup analysis, and five of them described the TCM identification typology as liver depression and qi stagnation types, so there was no significant heterogeneity, *I*^2^ = 0% < 50%, *P*=0.003, *Z* = 3.01, which was statistically significant. Three studies did not account for whether the included subjects were identified and staged, so the results were moderately heterogeneous, *I*^2^ = 53%, *P* < 0.00001, *Z* = 5.81, which was statistically significant. The final meta-analysis showed that *P*=0.56 > 0.1, *I*^2^ = 0% < 50%, and the fixed effects model was selected for analysis, which finally yielded RR = 1.19, 95% CI (1.13, 1.25), *P* < 0.00001, *Z* = 6.37. The efficacy of the qi-blood cotherapy method to improve the symptoms was better than that of the anti-anxiety-depression medicine, and the difference between the two groups was statistically significant, as shown in Figure 8.

### 3.6. Seattle Angina Questionnaire (SAQ) Scores

Two of the included studies [22, 23] compared the SAQ points by dimension, which included the degree of limitation of physical activity (PL), angina stability (AS), angina frequency (AF), treatment satisfaction (TS), and disease perception (DP). The results showed that the SAQ scores for each dimension improved after treatment in both groups. Compared with the control group, the qi-blood cotherapy group showed a significant improvement in AS (SMD = 0.65, 95% CI (0.29, 1.01), *P*=0.0004), AF (SMD = 0.64, 95% CI (0.11, 1.18), *P*=0.02), and TS (SMD = 0.95, 95% CI (0.25, 1.65), *P*=0.008). These results indicated that in terms of maintaining the stability of angina pectoris, reducing the frequency of attacks, and increasing the degree of patient satisfaction, the qi-blood cotherapy group had better results (see Table 2). There was no significant difference in physical limitations or disease perception between the two groups. We checked the selected studies and found that the herbal formulas were not identical, which may contribute to the heterogeneity. Moreover, the intervention duration was different, which may be a source of heterogeneity.

### 3.7. Incidence of Adverse Reactions

**Figure 9:**
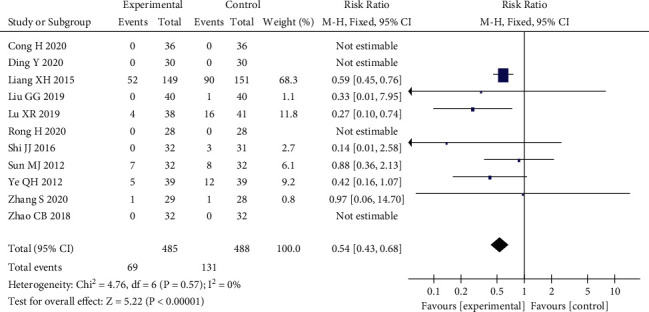
Meta-analysis forest plot of the incidence of adverse reactions.

Eleven of the included studies [11–13, 15–17, 20–24] reported the occurrence of adverse reactions, and the symptoms were mostly dry mouth, fatigue, dizziness, nausea, vomiting, abdominal pain, diarrhea, insomnia, and hepatic and renal impairment. The meta-analysis results showed that *P*=0.57 > 0.1, *I*^2^ = 0% < 50%, and there was no significant heterogeneity in these studies, so a fixed effects model analysis was selected, which finally yielded RR = 0.54, 95% CI (0.43, 0.68), *P* < 0.00001. The risk of adverse reactions in the qi-blood cotherapy group was significantly lower than that in the control group, and the difference between the two groups was statistically significant (see Figure 9).

### 3.8. Publication BIAS

**Figure 10:**
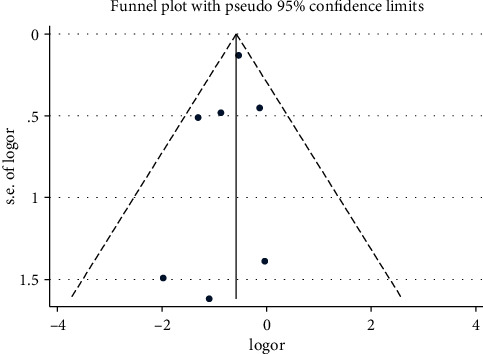
Publication risk of bias funnel plot.

**Figure 11:**
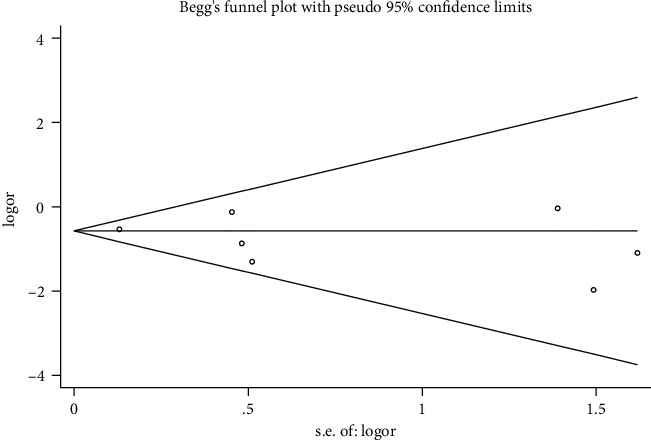
Graph of Begg's test results.

Stata 12.1 was used to assess the incidence of adverse reactions as indicators, and the indicators were plotted in a funnel plot to examine whether there was publication bias in the literature. It was concluded that both sides of the funnel plot were basically symmetrical, and Begg's test *P*=0.374 > 0.05, suggesting that there was no publication bias in the included studies and thus the results were reliable. The details are shown in Figures 10 and 11.

### 3.9. Quality of Evidence Grade Evaluation

The outcome indicators HAMA posttreatment score reduction, efficacy of TCM, and incidence of adverse reactions were supported by moderate evidence, and the HAMD posttreatment score reduction and SAQ score were supported by low-quality evidence. The essential factors were mainly the absence of allocation concealment, blinding, and high heterogeneity, as shown in Table 3.

## 4. Discussion

National and international studies have confirmed that cardiac-psychological comorbidity significantly increases the risk of death in patients after PCI [25] and that negative emotions accelerate the inflammatory response in the body and cause alterations in cardiac autonomic function, which in turn reinjures the coronary arteries, creating a vicious cycle. Moreover, they are often clinically overlooked by physicians because of overlapping or inconspicuous symptoms, and only 3.2% of patients are diagnosed, with less than half of those actually attending regular consultations [26]. Therefore, identifying it early, ensuring treatment compliance, and improving patients' quality of life are pressing challenges to be solved.

The harmonization of qi and blood originates from the Yellow Emperor's Classic of Internal Medicine. Harmony is also known as “concord,” which means “harmony is precious” and is called the “Harmony Method” in TCM treatment. Professor BS Xue, a master of Chinese medicine, believes that “loss of harmony” is the main pathogenesis of the disease [27] and emphasizes that heart disease is treated not only by the heart but also by the whole body. He has always advocated the idea of “harmonizing the disharmony to harmonize.” The pathogenesis of this disease is actually “qi-blood disharmony,” mainly manifested in the “heart loss of nourishment” and “liver loss of drainage.” MY Wang, a famous writer of the Qing Dynasty, said, “Fire cannot be born without wood, it must follow wood to follow it.” The doctor SD Chen tried heart-liver cotreatment and achieved satisfactory clinical results [28]. The heart and liver are physiologically interconnected through the meridians, and their functions complement each other, focusing on the operation of the blood vessels and the regulation of emotions. “The liver collects blood, and the heart carries it out.” The liver is well organized and relaxed, and the veins are fluid, which helps the heart carry blood; the heart can produce blood, the liver stores blood, and the blood is abundant, which moistens the liver and the heart veins. “Heart-liver harmony” is the basis of normal emotional activity. Heart malfunction, and the child's illness, affect the mother, resulting in liver draining function, qi dysregulation, depression, depression, anxiety, qi depression, and then all depression, qi stagnation, and blood stagnation and blood stasis, or qi stagnation and the collection of phlegm. If the liver wood does not reach, the qi stagnates and turns into fire, and the mother's illness affects the child, then insomnia, irritability, and other anxiety symptoms appear. Liver depression multiplies the spleen, causing deficiency due to realness, the spleen loses health, then the qi and blood biochemical lack a source, the heart loses its nourishment; or over time, it involves the kidney Yin, and eventually, the five viscera lose their harmony. In addition, PCI, as an invasive operation, will inevitably affect the balance of Qi, Blood, Yin, and Yang in the body while opening the problematic blood vessels. Based on the idea of harmonization, this study proposed the idea of “qi-blood harmony” for the treatment of negative emotions after PCI, which will have some significance in the management and prevention of difficult problems after the intervention.

The drugs that were used more frequently in the included studies were Radix Bupleuri (12 times), Paeoniae Radix Alba and Radix Paeoniae Rubra (10 times), licorice (10 times), Chuanxiong Rhizoma (8 times), Cyperi Rhizoma (8 times), Aurantii Fructus (7 times), Curcumae Radix (6 times), and Radix Salviae (6 times). Radix Bupleuri, the main herb for detoxifying the liver and relieving depression, mainly enters the liver and gallbladder meridians and is pungent, and modern pharmacological studies have found that it can alleviate negative emotions by inhibiting *γ*-aminobutyric acid in the central nervous system [29]. Paeoniae Radix Alba and Radix Paeoniae Rubra enter the liver and spleen meridians and are good at nourishing Yin and softening the liver; they are often used in combination with Radix Bupleuri to regulate the Qi and Blood, and the extract has analgesic and antidepressant effects. Modern studies have found that Chuanxiong Rhizoma can slow down the process of coronary atherosclerosis, dilate blood vessels, resist platelet aggregation, protect cardiomyocytes, reduce reperfusion injury [30], and enhance the antioxidant capacity of rat myocardial tissue [31]. Aurantii Fructus and Radix Bupleuri radix, one descending and one ascending, move Qi and relieve depression, and their active ingredients have the effects of antiatherosclerosis, protection of cardiomyocytes, antithrombotics, and inhibition of platelet aggregation. The herbs Curcumae Radix, Lilii Bulbus can help to relieve depression and tranquilize the blood, which can work together with Radix Bupleuri to regulate Qi and relax the liver. Licorice tonifies the heart Qi and benefits the Qi to restore the pulse. Radix Salviae enters the heart and liver blood division and is highly selective for the heart meridian; it is good at tonifying blood without leaving stasis and invigorating blood without harming the righteousness and can clear the heart and remove irritation. In combination with Paeoniae Radix Alba and Radix Paeoniae Rubra, it can remove stasis and generate new blood. Its main component, phenolic acid, has various protective effects, such as improving myocardial ischemia and hypoxia or injury, reducing blood viscosity, promoting myocardial microcirculation, and antiatherosclerosis. In contrast, Radix Salviae drink has anti-myocardial fibrosis and apoptosis effects, protects endothelial cells, and controls the release of inflammatory factors [32].

This study was based on Cochrane systematic evaluation principles for the clinical efficacy and safety of treatments with negative affect after qi-blood harmonization treatment, and the quality of evidence was scored by GRADE profiler 3.6 for outcome indicators in the included studies. According to the results, compared with conventional anti-anxiety and depression medications, the qi-blood cotherapy herbal group had beneficial effects for clinical symptoms (RR = 1.19, 95% CI (1.13, 1.25), *P* < 0.00001, *Z* = 6.37), maintaining the stability of angina pectoris (SMD = 0.65, 95% CI (0.29, 1.01), *P*=0.0004), increasing the degree of patients' satisfaction (SMD = 0.95, 95% CI (0.25, 1.65), *P*=0.008) reducing the frequency of attacks (SMD = 0.64, 95% CI (0.11, 1.18), *P*=0.02), and the incidence of adverse effects (RR = 0.54, 95% CI (0.43, 0.68), *P* < 0.00001). This result was consistent with the results of meta-analysis studies by Liu [33]and Lu [34].

The HAMA and HAMD scores decreased significantly in both groups, and there was no statistically significant difference between the Chinese herbal medicine group and the Western medicine group in terms of reduction of the HAMA score (RR = 1.03, 95% CI (0.95, 1.12), *P*=0.40, *Z* = 0.83), and the efficacy of the treatments were comparable. In terms of reduction of the HAMD rating scale, a sensitivity analysis revealed the study by Liang affected the results. After excluding the results of Liang, the qi-blood cotherapy herbal group was statistically superior to the western medicine group in reducing the HAMD score (RR = 1.12, 95% CI (1.01, 1.24), *P* < 0.05). The results of HAMA in Liang differed from the findings of the above authors, mainly because most authors used the HAMA or HAMD posttreatment scores compared directly with controls for continuous variable analysis, while Xiang chose the difference between the pre- and posttreatment scores as the outcome indicator. Because the scoring of the scale is subjective, the results of scoring would vary among different people, and there were studies in which the scores of the control group after treatment were lower than those of the experimental group, but the degree of improvement was less than that of the experimental group. There is a risk of bias in the direct analysis of the posttreatment results, so the difference between the before and after improvement may be more accurate than the direct comparison of the posttreatment scores.

In terms of publication bias, funnel plotting for the incidence of adverse reaction outcome indicators and using Begg's test proved that there was no significant publication bias in the literature included in this study and that the study results were reliable. In the treatment of negative emotions after PCI, anti-anxiety and depression, Western drugs only focus on improving anxiety and depression, with no significant improvement in coronary artery disease itself, as well as symptoms such as postoperative recurrent angina, stenosis or palpitations, and restlessness, and inevitable drug side effects. In contrast, the qi-blood cotreatment method improves the adverse mood, and its efficacy was not significantly different from or even better than that of Western drugs. It also focuses on harmonizing the Qi, Blood, and Yin and Yang of the five organs, multidimensional, multilinked, and multitargeted treatment of coronary heart disease [35], which was beneficial in relieving many uncomfortable symptoms of the coronary heart disease itself and postoperative symptoms, and could reduce the incidence of adverse reactions, with one symptom on one side, with individualized identification according to the patient differences, fully demonstrating the superiority of TCM treatment.

The results of this study combined with the GRADE evaluation suggest that the outcome indicators HAMA posttreatment score reduction efficiency, efficacy of TCM, and the incidence of adverse reactions were supported by moderate evidence, and the HAMD posttreatment score reduction and SAQ scores were supported by low-quality evidence. The results obtained were reliable, providing an evidence-based reference for the direction of clinical treatment, and that the overall efficacy and safety of qi-blood cotherapy based on the idea of harmonization was highly beneficial in patients after PCI, which is worthy of further clinical promotion.

### 4.1. Limitations

However, there were some limitations in this study: (1) few high-quality, multicentre studies were included; most trials did not describe randomization methods in detail, and there was a risk of “pseudorandomization” in many trials. No trials mentioned allocation concealment methods, and only two studies were blinded, which may have resulted in selection and implementation bias. Only a few databases were searched, and the study dimension was insufficient. (2) Long-term follow-up was not mentioned in the included studies, and the follow-up of the patients after PCI was not available, which made it difficult to assess the long-term efficacy of TCM, the patients' later quality of life, and the recurrence of MACE, and it is recommended to extend the observation period. (3) The scale evaluation was susceptible to subjective factors and lacked standard objective physical and chemical indicators. (4) The outcome indicators were not standardized and unified; for example, a few studies used anxiety and depression scales (SAS, SDS, GAD-7, and PHQ-9) and the Short Form of Health Survey (SF-36) was only used by a single study, and this did not allow for combined analysis of these outcomes. Therefore, in the future, we should focus on rigorous methodology and conduct a multicentre, large sample, high-quality randomized double-blind RCT with more TCM characteristics for validation.

## Figures and Tables

**Table 1:** Basic characteristics of the included studies.

Study	Year	Sample size (T/C)	Mean age (years, $\bar{x}\pm s$)		Interventions		Treatment (weeks)	Ending indicators
			T	C	T	C		
Cong [11]	2020	72 (36/36)	NA	NA	Yangxin decoction	Deanxit	4	①②③
Ding [12]	2020	60 (30/30)	52.43 ± 7.74	54.76 ± 9.20	Baihetiaolv decoction	Deanxit	4	①②
Lu [13]	2019	79 (38/41)	64.13 ± 6.72	63.12 ± 7.55	Jieyushugantongmai decoction	Deanxit	2	③④
Qi [14]	2017	90 (45/45)	51 ± 9.4	54 ± 10.6	Jieyutongmai granules	Deanxit	2	②
Liang [15]	2015	300 (149/151)	76 ± 4	77 ± 5	Shuganjieyu decoction	Deanxit	6	①④
Ye et al. [16]	2012	78 (39/39)	67.0 ± 8.0	68.0 ± 7.0	Shuganjieyu capsules	Paroxetine	6	①②③④
Liu et al. [17]	2019	80 (40/40)	48.45 ± 10.35	48.22 ± 11.23	Yuzheng decoction	Deanxit	2	①④
Liu [18]	2015	183 (92/91)	NA	NA	Yuzheng decoction	Deanxit	8	①
Wang [19]	2016	60 (30/30)	NA	NA	Chaihushugan decoction	Deanxit	14	③
Rong [20]	2020	60 (30/30)	55.10 ± 7.59	56.47 ± 6.32	Chaihuwendan decoction	Deanxit	4	①②
Zhang [21]	2020	60 (30/30)	57.86 ± 8.07	57.31 ± 7.63	Chaiguilongmu decoction	Deanxit	4	①②④
Zhao [22]	2018	64 (32/32)	60.22 ± 5.96	56.88 ± 7.25	Shuganjieyutongmai decoction	Escitalopram	16	①③⑤
Shi [23]	2016	68 (34/34)		58.55 ± 1.52	Jieyutongmai decoction	Escitalopram	8	③④⑤
Sun [24]	2012	60 (30/30)	58.2	56.1	Jieyutongbi decoction	Escitalopram	6	③④

Notes: T, intervention groups; C, control groups; NA, did not mention; ① efficacy of TCM evidence; ② HAMA pre- and posttreatment score reduction efficiency; ③ HAMD pre- and posttreatment score reduction efficiency; ④ incidence of adverse events; and ⑤ SAQ.

**Table 2:** Analysis of SAQ subgroups.

SAQ	Heterogeneity		Effect value	
	*I * ^2^ (%)	*P*	SMD [95% CI]	*P*
PL	69	0.07	0.50 [−0.14, 1.13]	0.13
AS	0	0.37	0.65 [0.29, 1.01]	0.0004^*∗*^
AF	55	0.14	0.64 [0.11, 1.18]	0.02^*∗*^
TS	72	0.06	0.95 [0.25, 1.65]	0.008^*∗*^
DP	73	0.06	0.20 [−0.48, 0.87]	0.57

^*∗*^ *P* < 0.05, there was statistical significance.

**Table 3:** Evaluation of GRADE evidence quality.

Outcome indicators	95% CI	Risk of bias	Inconsistency	Indirectness	Imprecision	Publication bias	Upgrade quality of evidence	Quality
HAMA post treatment score reduction	RR 1.03 (0.95, 1.12)	serious①	No	No	No	Undetected	None	Moderate
HAMD post treatment score reduction	RR 1.05 (0.99, 1.10)	serious①	serious②	No	No	Undetected	None	Low
Efficacy of TCM	RR 1.19 (1.13, 1.25)	serious①	No	No	No	Undetected	None	Moderate
SAQ	SMD 0.58 (0.33, 0.84)	serious①	serious②	No	No	Undetected	None	Low
Incidence of adverse reactions	RR 0.54 (0.43, 0.68)	serious①	No	No	No	Undetected	None	Moderate

SMD is the standardized mean difference; RR is the relative risk; ① absence of allocation concealment and blinding; ② the *p* value of the heterogeneity test was <0.1, and *I*^2^ >50%.

## Data Availability

The data used to support this systematic review are from previously reported studies, which have been cited within the article.

## Abbreviations

CHD: Coronary heart disease
PCI: Percutaneous coronary intervention
RCTs: Randomized controlled trials
TCM: Traditional Chinese medicine.
